# Association between dental caries and obesity among Libyan schoolchildren during the armed conflict in Benghazi

**DOI:** 10.1186/s12903-023-02728-2

**Published:** 2023-01-25

**Authors:** Entesar Aoun, Lamis Ballo, Sara Elhabony, Arheiam Arheiam

**Affiliations:** Department of Dental Public Health and Preventive Dentistry, Faculty of Dentistry, University of Benghazi, Benghazi, Libya

**Keywords:** Dental caries, Obesity, Children, Libya, Observational study

## Abstract

**Background:**

Dental caries and Obesity in children are issues of public health concern. Even though researching the relationship between these two noncommunicable diseases has been conducted for many years, the results remain equivocal. This paper aimed to examine the association between dental caries and obesity among 12-year-old schoolchildren living in war-affected environment in Benghazi.

**Methods:**

A secondary analysis of a cross-sectional study was conducted to determine the prevalence of caries among 12-year-old school children in Benghazi in 2017 during the armed conflict that affected the city. The data extracted for the analysis included sociodemographic of the participants (gender, maternal education and school type), caries experience (DMFT index), and anthropometric measures (height in cm, weight in kg, BMI and Z score for BMI). Comparisons of anthropometric measures were conducted according to caries experience. Linear regression models were developed to determine the association between Body Mass Index and Z score as outcome variables, caries as an explanatory variable, and covariates (gender, maternal education and school type). Beta coefficient (β) and 95% confidence intervals were calculated. All statistical tests were conducted at *p* ≤ 0.05.

**Results:**

There were 782 children with a mean (SD) BMI of 20.7 SD5.09 and an average z (SD) score of 0.56 SD1.51. Also, 159 (20%) children had obesity. No significant association was observed between caries and anthropometric measures. However, higher BMI was observed in children from a private school (*p* ≤ 0.001***), females (*p* ≤ 0.001***) and self-reported regular sugary drinks consumers (*p* ≤ 0.001***).

**Conclusion:**

The present study shows no significant association between dental caries and anthropometric measures. However, the study findings support the notion of tackling sugar intake as a common risk factor for caries and obesity, which should be encouraged in the Libyan culture.

## Introduction

Libya, a war-torn North African country, that since the year 2011 endured several military conflicts and a financial crisis. Benghazi, the second largest city and the capital of Libyan east province, was locked in armed conflict that lasted three years since May 2014 and has resulted in life hardships affecting social and behavioural aspects of life [1]. One of the health-related consequences of conflict was the drop in sugar consumption among Libyan children, an undebated common risk factor for many health conditions [2]. Obesity and caries are global public health problems that have significant burdens, negatively impact the quality of life and share sugar consumption as a common risk factor [3]. The available data suggests that 16.9% of children aged five or younger and 6.1% of children aged between 10 and 18 were obese. Obesity among Libyan children and adolescents is increasing due to adopting a western diet rich in fat and sugars [4]. Furthermore, the impact of decreased sugar intake during the Libyan conflict has been demonstrated in a relatively recent study showing a concurrent decrease in caries and sugar intake compared to pre-conflict higher prevalence of dental caries [1]. However, searching literature indicated that no recent studies had been conducted to assess obesity and its risk factors among Libyan children during the Libyan conflict. Lacking pre-conflict obesity data makes it impossible to assess whether the prevalence of obesity is affected by decreased sugar intake during the conflict, in spite of the facts that sugar intake is a common risk factor for caries and obesity [5]. Moreover, both conditions are multifactorial, with an interplay between biological, genetic, socioeconomic, cultural, dietary, environmental, and lifestyle risk factors [6]. Therefore, it is unclear to what extent the reduced sugar intake during the conflict has affected the distribution of obesity.

The relationship between caries and obesity has received much attention in recent years. However, the evidence from several systematic is equivocal [7–13], which is partly attributed to inconsistencies in assessment and measurements of both conditions [10, 14]. More importantly, the previous reviews highlighted the need to adjust the caries-obesity relationship for the social environment and sugar intake, which are well-known common risk factors for both caries and obesity [15, 16]. The theoretical model of social determinants of health have suggested that health outcomes are influenced by behavioural determinants, which in turn are influenced by wider social determinants [5]. This theoretical explanation allows for policy development and planning of public health interventions which should be tailored to the social environment and behaviours. For example, there is evidence that associations between dental caries and obesity in children vary by country-level income [13]. In addition, several studies conducted in low-Middle income countries reported conflicting findings regarding the association between caries and obesity [17–22]. For example, recent studies conducted in India [23] and Egypt [24] demonstrated variations in caries-obesity relationship and sugar consumption. It is, therefore, helpful to assess the association between caries and obesity in different environmental and social contexts where there are variations in obesogenic behaviours, dietary habits, and cultural habits. Given this, the present study aimed to investigate the association between caries and obesity among schoolchildren living in a war-affected environment in Benghazi and to evaluate the role of family socioeconomic status and sugar consumption in explaining this association.

## Methods

Permission to use the primary data was obtained from the Department of Dental Public Health at the University of Benghazi. Research Ethics Committee approved the study at the Faculty of Dentistry, University of Benghazi (Ref No: UOB-053). The reporting of the study followed the statement outlined in strengthening the reporting of observational studies in Epidemiology (STROBE).

### Study design and setting

A secondary analysis was conducted on primary data collected in 2017, during wartime in Benghazi, as part of a cross-sectional survey of 12-year-old school children. Benghazi is the second largest city in the country, with nearly a million inhabitants from different families and tribes. Hence, this population is deemed representative of all Libyans. The education system is comprised of the public and private sectors. The ministry of education divided the city of Benghazi into eight administrative districts based on the geographic distribution of the population and representing urban and suburban populations.

### Study population

The participants were recruited from public and private schools in Benghazi. It was estimated that 13,000 12-year-old schoolchildren were registered in 2016/2017. The students were distributed over 267 schools, and around 75% were enrolled in public schools. A study sample of 1200 pupils was selected using a two-stage random sampling strategy. In the first stage, a random sample of 40 public and private schools were selected proportionally from 8 administrative districts in Benghazi. The average number of students in each classroom was 30. Therefore, one classroom was selected randomly from each school. Informed consent was first sought from the parents and sent to them through the school administrator's office. A detailed description of the original sample calculations has been provided elsewhere [1]. The aim of the study was explained to the children, and verbal assent was implied by accepting to attend the dental examination. A total of 1134 parents returned signed consent forms and completed questionnaires usable for data analysis. Only Libyan children who had lived in the city of Benghazi since their birth were recruited for the study. Children diagnosed with a mental or physical disability, undergoing orthodontic treatment, with hypoplastic teeth or moderate to severe dental fluorosis were excluded.

For the current study, incomplete data in which the date of birth, height, or weight was missing was removed. As a result, 782 participants were included in the analysis. Post-hoc power analysis demonstrated that this sample size is sufficient to assess the association between obesity and caries at 99% power at statistical significance of 0.05, and 95% confidence intervals.

### Measurements

#### Dental caries measurements

Dental caries lesions were assessed according to World Health Organization (WHO) diagnostic criteria (at the dentine level) by using the DMFT index [1]. The dental examination was conducted for all participants in a separate classroom under natural daylight while the participant was seated on an ordinary chair using disposable diagnostic kits. Three dentists were trained and calibrated to carry out the clinical dental examinations. Kappa coefficients were 0.88–0.96 for inter-examiner and intra-examiner reliability. The training sessions were provided at the Department of Pediatric Dentistry, University of Benghazi, before commencing the data collection.

#### Anthropometric measurements

The investigators conducted the anthropometric measurements. Height was measured in cm, with the child standing without shoes using a portable stadiometer. Weight in kg was measured using a pre-calibrated digital Seca scale, with children wearing light clothes and no shoes. The measurements for height and weight were taken to the nearest 0.1 cm and 0.1 kg, respectively. BMI and BMI-adjusted z-scores for Age and gender (BAZ) were computed based on WHO growth references data for children aged 5–19 years using the WHO Anthro-plus software program.[2, 25].

### Questionnaire

The questionnaire was developed from previous studies and guided by the study's research questions [1]. Before data collection, the questionnaire was tested for clarity and readability among a group of dental patients and schoolchildren. The questionnaire collected sociodemographic data (children's date of birth, gender and parental educational level). In addition, the questionnaire included items related to oral health-related behaviours (for example: whether they brushed their teeth regularly) and dietary habits such as timing and frequency of sugar intake (never, sometimes, once per day, twice or more per day). The questionnaire was paper based and handed out by a trained assistant to the children to complete with the assistance of their parents.

### Data management and analysis

The data extracted for the present study were how frequently sugary drinks and foods are consumed, which was then dichotomized as infrequent (never and sometimes), and frequent (once per day, twice or more per day), caries as present (DMFT ≥ 1) or absent (DMFT = 0), maternal education (less than university vs. university or higher), and school type (public vs. private) [26]. The third model of WHO Anthro-Plus was used to compute BMI and BAZ from the original data. The participants were grouped into four categories: low weight, average weight, overweight, and obese, following the cut-off points of <  − 2 SD, ≥  − 2 SD— + 1 SD, >  + 1 SD— + 2 SD, and >  + 2SD z-scores, respectively [27]. The data was then uploaded to SPSS 25 software for analysis. Descriptive statistics were conducted to summarise the sample profile, regular dietary habits, height, weight, BMI and Z-scores. Bivariate comparison using the independent sample t-test was used to compare anthropometric measures (height and weight), BMI and BAZ according to children’s school type, caries experience, maternal education and sugar intake. Multilevel multiple linear regression analysis was used to assess the association between BMI and BAZ (dependent variable) and fixed effect factors including level 1 (child) factors: caries experience, gender, maternal education, sugar intake and level 2 (school) factors: public versus private). The analysis was built on the common risk approach for tackling non-communicable diseases. Obesity and caries share common risk factor such as sugar intake and social position in the community [5, 6]. Therefore, association was examined between caries (as predictor) and obesity (as an outcome) and social characteristics as well as sugar intake were included as confounders. An unconditional model (without fixed effect variables) was first created as baseline. This was followed by three conditional models including child, school and combined child-school factors as fixed effects. Improvement in model fit due to the added fixed effect variables was assessed using model deviance (− 2LL) and covariance parameter estimates. Adjusted regression coefficients (B) and their respective 95% confidence intervals (CI) were estimated and reported. A two-way ANOVA was conducted to examine the effect of school type and sugar consumption on BAZ and caries experience (DMFT). All statistical tests were conducted with a *p* value set at ˂0.05.

## Results

Data from 782 children aged 12 years were included in the analysis. Most of them were from public schools (591, 75.6%). More than half of the participants were males (422, 54%), and more than half did not have caries (437,55.9%). Slightly less than half of the mothers (329, 47.7%) did not attain a university education (Table 1). Average weight was observed in the majority of the participants (456, 58%), whereas obese children represented (159, 20%) of the participants and a small proportion (43, 6%) were underweight.

**Table 1:** Comparisons of height, weight, BMI and Z-scores by caries experience, sugar consumption, school type and maternal education (N = 782)

Variables	Overall N (%)	Height Mean (SD)	Weight Mean (SD)	BMI Mean (SD)	Z score Mean (SD)
		146.1 (9.59)	44.4 (12.93)	20.7 (5.09)	0.56 (1.51)
*Sugary drinks*					
Regular	325 (41.6)	145.77 (9.49)	46.41 (13.60)	21.26 (5.37)	0.502 (1.5)
Irregular	457 (58.4)	146.37 (9.66)	42.27 (11.81)	20.08 (4.69)	0.508 (1.5)
*p* value		0.023*	≤ 0.001***	≤ 0.001***	≤ 0.001***
*Caries*					
Present	345 (44.1)	145.26 (9.98)	43.01 (11.21)	20.42 (4.71)	0.49 (1.48)
No caries	437 (55.9)	146.79 (9.23)	45.44 (14.05)	20.90 (5.36)	0.52 (1.52)
*p* value		≤ 0.001***	≤ 0.001***	0.001**	0.479
*School type*					
Private	191 (24.4)	145.9 (8.78)	45.97 (14.70)	21.36 (5.28)	0.62 (1.48)
Public	591 (75.6)	146.3 (10.4)	43.05 (11.03)	20.11 (4.85)	0.40 (1.52)
*p* value		0.116	≤ 0.001***	≤ 0.001***	≤ 0.001***
*Gender*					
Male	422 (54)	144.83 (9.84)	42.27 (11.8)	20.08 (4.69)	0.51 (1.51)
Female	360 (46)	147.32 (9.21)	46.41 (13.6)	21.26 (5.37)	0.50 (1.50)
*p* value		≤ 0.001***	≤ 0.001***	≤ 0.001***	0.873
*Maternal education*					
Less than University	329 (47.7)	146.23 (9.98)	44.40 (13.17)	20.66 (5.19)	0.48 (1.58)
University or higher	453 (52.3)	146.00 (9.56)	44.81 (13.26)	20.92 (5.25)	0.59 (1.42)
*p* value		0.446	0.280	0.090	0.014*

Independent sample t test was used to compare height, weight, BMI and Z-score across study subgroups. Height in cm, weight in kg

*≤ 0.05, **≤ 0.01, ***≤ 0.001, *SD* standard deviation

Table 1 shows summary statistics on anthropometric measures and comparisons according to the participants' gender, maternal education, caries, school type and consumption of sugary drinks. The average BMI was 20.7 (SD = 5.09), and the average z-score was 0.56 (SD = 1.51). Comparisons of anthropometric measures demonstrated a statistically significant difference in BMI and weight when compared by sugar intake frequency, school type and gender. Regular consumers of sugary drinks had higher average body weight (*p* ≤ 0.001) and BMI (*p* ≤ 0.00 compared to irregular consumers. Children who studied in private schools have higher body weight (*p* ≤ 0.001) and BMI (*p* ≤ 0.001) than those in public schools. Females had higher height (*p* ≤ 0.001), body weight (*p* ≤ 0.001) and BMI (*p* ≤ 0.001) than males.

Results of multilevel linear regression analyses (Table 2) revealed non statistically significant and weak associations between DMFT and BAZ when adjusted for child level factors [B = 0.02, 95% CI (− 0.04, 0.10); *p*(0.56)] and both child level and school level factors [B = 0.03, 95% CI (− 0.05, 0.10); *p*(0.47)]. No statistically significant association was reported between BAZ and child or school level factors.

**Table 2:** Association between BAZ and caries experience (DMFT) controlling for confounders using multilevel multiple linear regression analysis

	Unconditional	Conditional 1 (with fixed effect factors) B (95% CI); [*p* value]	Conditional 2 (with fixed effect factors) B (95% CI); [p value]	Conditional 3 (with fixed effect factors) B (95% CI); [p value]
*Child factors*				
DMFT	–	0.02 (− 0.04, 0.10); [0.56]	–	0.03 (− 0.05, 0.10); [0.47]
Gender: Male versus Female	–	0.06 (− 0.16, 0.28); [0.61]	–	0.04 (− 0.19, 0.23); [0.74]
Maternal education Less than University VS Univrsity	–	0.08 (− 0.03, 0.43); [0.47]	–	0.04 (− 0.20, 0.27); [0.75]
Sugary drinks Infrequent versus frequent	–	0.20 (− 0.14, 0.31); [0.08]	–	0.20 (− 0.04, 0.34); [0.09]
*School factors*				
School type Public versus private	–	–	0.21 (− 0.03, 0.47); [0.08]	0.26 (− 0.01, 0.53); [0.06]
− 2LL	2865.6	2521.7	2862.5	2518.1
Covariance parameter: estimate (SE)	2.29 (0.12)	2.27 (0.12)	2.29 (0.12)	2.26 (0.12)

Conditional 1: including child level factors: DMFT and confounders (gender, maternal education and consumption of sugary drinks)

Conditional 2: including only school level factors (public vs. private, as confounder)

Conditional 3: including both child and school level factors

Table 3 shows the results of multilevel linear regression analysis for BMI as dependent variable. No statistically significant association was reported between DMFT and BMI, when adjusted for child level factors [B =  − 0.08, 95% CI (− 0.32, 0.16); *p*(0.58)] and both child level and school level factors [B =  − 0.06, 95% CI (− 0.30, 0.18); *p*(0.62)]. Significant associations between BMI and social and behavioural factors were reported in separate models for child level factors and school level factors. Higher BMI was reported among frequent consumers of sugary drinks [B = 0.83, 95% CI (0.05, 1.61); *p*(0.04)] females [B = 0.89, 95% CI (0.13, 1.67); *p*(0.02)], and private schools [B = 1.24, 95% CI (0.43, 2.05); *p*(0.003)]. The association remained statistically significant even after combining child level and school level factors in one model. Higher BMI was associated with frequent consumption of sugary drinks [B = 0.80, 95% CI (0.03, 1.58); *p*(0.04)] females [B = 1.01, 95% CI (0.25, 1.77); *p*(0.01)], and private schools [B = 1.43, 95% CI (0.52, 2.33); *p*(0.002)].

**Table 3:** Association between BMI and caries experience (DMFT) controlling for confounders using multilevel multiple linear regression analysis

	Unconditional	Conditional 1 (with fixed effect factors) B (95% CI); [*p* value]	Conditional 2 (with fixed effect factors) B (95% CI); [*p* value]	Conditional 3 (with fixed effect factors) B (95% CI); [*p* value]
*Child factors*				
DMFT	–	− 0.08(− 0.32, 0.17); [0.53]	–	− 0.06 (− 0.30, 0.18); [0.62]
Gender: Male versus female	–	0.89 (0.13, 1.67); [0.02*]	–	1.01 (0.25, 1.77); [0.01**]
Maternal education Less than University VS University	–	0.06 (− 0.70, 0.83); [0.87]	–	− 0.18 (− 0.96, 0.50); [0.64]
Sugary drinks Infrequent versus frequent	–	0.83 (0.05, 1.61); [0.04*]	–	0.80 (0.03, 1.58); [0.04*]
*School factors*				
School type Public versus private	–	–	1.24 (0.43,2.05); [0.003**]	1.43 (0.52, 2.33); [0.002**]
− 2LL	4731.6	4197.5	4722.6	4187.9
Covariance parameter: estimate (SE)	24.85 (1.25)	25.67 (1.38)	24.56 (1.24)	25.32 (1.36)

*p ≤ 0.05, **p ≤ 0.01

Conditional 1: including child level factors: DMFT and confounders (gender, maternal education and consumption of sugary drinks)

Conditional 2: including only school level factors (public vs. private, as confounder)

Conditional 3: including both child and school level factors

Table 4 shows the results of two-way ANOVA. There was a statistically significant interaction between the effects of school type and sugar intake on Z score (*p* = 0.001) and caries experience (*p* = 0.001). Children from private schools who are frequent consumers of sugars had the highest average Z-score (0.76, SD = 1.47). On the other hand, public school children and infrequent consumers of sugars had the least Z-score (0.33, SD = 1.52). On the contrary, public schools have higher DMFT regardless of sugar consumption, though the lowest score was among infrequent sugar consumers in private schools (0.63, SD = 1.15).

**Table 4:** Two-way ANOVA analysis of the effect of school type and sugar intake on obesity and caries

SCHOOL	Sugary drinks	BAZ		DMFT	
		Mean	SD	Mean	SD
Private	Infrequent	0.42	1.46	0.63	1.15
	Frequent	0.76	1.47	0.91	1.31
Public	Infrequent	0.33	1.52	0.98	1.56
	Frequent	0.45	1.52	1.00	1.54
*p* value for school-sugar interaction		0.001**		0.001**	

**p ≤ 0.01

## Discussion

The present study sought to investigate the association between caries and obesity among schoolchildren during wartime and to evaluate the role of family socioeconomic status and sugar consumption, as common risk factors, in explaining this association. The data analysis indicates that caries and obesity were negatively associated. However, after controlling for common risk factors, this association became non-statistically significant when obesity was measured using BMI and changed in direction when BAZ was used as an obesity measure. Similar findings have been reported in a recent US study which concluded that the caries-obesity relationship is influenced by how they are measured and attenuated by common risk factors [14]. In addition, a negative association between dental caries and BAZ has been observed in studies conducted in Bangladesh [28] and Saudi Arabia [29]. Taken together, these observations, while explaining the inconclusive and conflicting findings reported in several reviews regarding the association between obesity and caries, it highlights the need to standardize the measurement of caries and obesity in future studies.

The present study’s findings support the role of common behavioural and social risk factors in influencing the association between caries and obesity. Although ecological data of Libyan school children living in the war environment linked reduced sugar intake to lower caries levels [1], high sugar intake is a well-documented cause of caries and obesity. Therefore, the positive association between sugar intake and obesity, observed in the present study, is a reflection of individual intake and should be by no means confused with per capita consumption of sugars during wartime. Evaluating the impact of reduced per-capita sugar intake during the conflict on obesity was not possible in our study due to a lack of comparable pre-conflict data on obesity among Libyan school children. However, a high sugar intake can indicate unhealthy behaviours such as increased consumption of fast foods, which are energy-dense and ultra-processed [30] and often combined with adopting a more sedentary lifestyle [31]. These behaviours are well-recognized as crucial drivers of increased obesity. However, further research is needed to understand the dietary habits of the Libyan culture and its association with obesity to uncover different pathways that might be at play for obesity among Libyan schoolchildren.

In the present study, higher BMI and BAZ were observed in children studying in private schools compared to their peers from public schools. This observation supports a moderating role of the social environment in the association between obesity and caries [32]. On the other hand, children from private schools had higher BAZ and lower DMFT score than those from public schools, and these scores vary according to the reported sugar consumption. One possible explanation for social variations in obesity and dental caries might be the differences in dietary habits, self-hygiene and lifestyle across the socioeconomic spectrum and living environments [33]. Put simply, it could be the case that children from higher income families (indicated by private schools) are capable of maintaining oral hygiene and has better access to dental care, while also having more access to energy-dense fast foods and video games that promote a sedentary lifestyle. So, their social position increases their risk of obesity and decreases their risk of caries, unlike their peers from low-income families. However, this assumption needs further investigation in future research using a more controlled study design. Nevertheless, the current study supports the notion that obesity and caries risk are not directly related; instead, common risk factors drive the development of both conditions. Furthermore, these factors are influenced by wider social determinants [14, 34].

To the authors' best knowledge, the present study was the first to assess the relationships between dental caries and anthropometric measures among Libyan adolescents. Our study provides baseline epidemiological data for both investigating trends of obesity and its association with dental caries in the future. The current study showed that a fifth of the study participants were classified as obese, which is way higher than what was previously estimated among Libyan children [4]. This finding, while confirming the projected increase in obesity in Libya due to adopting a more westernized diet, also chimes with the alarming global increase in obesity in children [35]. The increased prevalence of obesity in the Libyan population is expected in light of the increased adoption of poor dietary habits and sedentary lifestyles [4]. However, comparing the levels of obesity with that reported in other studies should be approached with caution because of differences in data collection methods and obesity assessment. Nevertheless, our findings highlight the urgent need to develop strategies and policies to tackle obesity among adolescents, especially since it is expected that sugar intake will increase after the armed conflict. However, there are some limitations in the present study which need attention. Firstly, the study is based on a secondary data analysis, making it impossible to control the variables used in the primary research. For example, we cannot rule out several potential risk factors for obesity, such as birth weight, breastfeeding in the infant stage, long-term dietary habits, family function, and a family history of obesity [3, 4, 36]. In addition, the primary data were collected using a cross-sectional design that does not allow for exploring causal relationships [37]. Moreover, the original sample calculation did not consider the design effect for clustered sample. Although a multilevel analysis was conducted considering school clusters, we acknowledge this as a limitation that should be avoided in future research. Therefore, results obtained in this research should be verified in future studies with more robust methodologies to inform interventional strategies in the population. Moreover, the primary research used the WHO criteria, which assessed caries at the dentin level; hence, the full caries spectrum was not measured. Therefore, further studies are needed using a comprehensive assessment of the dental caries status of schoolchildren using the International Caries Detection and Assessment System (ICDAS) [38].

## Conclusion

The present study found no direct association between caries and obesity among 12 year-old school children when the obesity was measured as BAZ and control for social position and sugar intake. Although obesity and caries appeared to have no direct relationship, both conditions share common risk factors, which supports applying a common risk approach to health promotion by emphasizing efforts to increase awareness about sugar's role in obesity and dental caries.

## Data Availability

The datasets used and analyzed during the current study are available from the corresponding author on reasonable request.

## Abbreviations

BMI: Body mass index
BAZ: BMI-adjusted z-scores for age and gender
DMFT: Decay-missing-filled teeth index
ICDAS: International Caries Detection and Assessment System
